# Patient interpretations of patient-reported outcome measures to assess bowel urgency: qualitative interviews in ulcerative colitis

**DOI:** 10.1186/s41687-024-00733-9

**Published:** 2024-05-31

**Authors:** Vipul Jairath, Theresa Hunter Gibble, Richard Moses, Brittany Klooster, Leighann Litcher-Kelly, Marisa Walker, Madison C Bernstein, Kaelyn Rupinski, Megan McLafferty, Simon Travis, Marla Dubinsky

**Affiliations:** 1 Departments of Medicine, Schulich School of Medicine and Dentistry, Western University, London, Canada; 2 Department of Epidemiology and Biostatistics, Western University, London, Canada; 3 Eli Lilly and Company, Indianapolis, IN USA; 4 Adelphi Values LLC, Boston, MA USA; 5 University of Oxford, Oxford, UK; 6 Icahn School of Medicine, New York, NY USA

## Abstract

**Objectives:**

Bowel urgency is an impactful core symptom of ulcerative colitis (UC). Patient-reported outcome (PRO) questionnaires have been developed and used to assess the patient experience of this important symptom. The objective of this paper is to present evidence from qualitative research conducted to support the use and interpretation of select PRO questionnaires to assess bowel urgency related to the UC patient experience.

**Methods:**

Qualitative interviews were conducted with ten adults with a clinician-confirmed diagnosis of moderately to severely active UC. Interviews aimed to document patient interpretation of modified recall periods for the Urgency Numeric Rating Scale (Urgency NRS), two global assessments (i.e., the Patient Global Impression of Severity [PGIS] and Patient Global Impression of Change [PGIC]), and four items (Items 11, 16, 23, and 26) of the Inflammatory Bowel Disease Questionnaire (IBDQ), and explore the patient perspective of meaningful change on these questionnaires.

**Results:**

Both modified Urgency NRS versions (with 7-day or 3-day recall period) were interpreted as intended by most patients (≥ 88.9%), and slightly more than half of patients (60.0%) reported that the 7-day recall period was more relevant to their bowel urgency experience. Patients reported thinking of bowel urgency (≥ 80.0%) or bowel urgency-related accidents (70.0% of patients) when interpreting the global assessments and IBDQ items. Most patients reported a 1- to 3-point change as the smallest meaningful improvement that would be meaningful on the Urgency NRS (similar to findings on other questionnaires).

**Conclusion:**

Adults with UC can understand and respond to the Urgency NRS with modified recall periods (i.e., 7-day or 3-day), interpret the conceptual content of the PGIS, PGIC, and select IBDQ items to be inclusive of bowel urgency and bowel urgency-related accidents, and select answers representing meaningful improvements on the Urgency NRS, PGIS, PGIC, and IBDQ item response scales. These results further contribute patient-centered data to existing UC and bowel urgency research.

**Supplementary Information:**

The online version contains supplementary material available at 10.1186/s41687-024-00733-9.

## Introduction

Inflammatory bowel disease (IBD) is a chronic idiopathic immune disorder characterized by uncontrolled and relapsing inflammation of the gastrointestinal tract and encompasses two major subtypes, ulcerative colitis (UC) and Crohn’s disease. UC is characterized by continuous inflammation of the innermost lining of the colon and the rectum. Within the United States, the prevalence of UC has been reported as 245.3 cases per 100,000 persons [1]. The primary symptoms of UC include frequent diarrhea with blood or pus in the stool, abdominal pain or discomfort, urgent need to have a bowel movement [2], fatigue, nausea, loss of appetite, weight loss, fever, and anemia [1, 3–5]. Bowel urgency, the sudden or immediate need to have a bowel movement, is a particularly bothersome and impactful symptom [2, 6–8]. Bowel urgency has been reported by patients as impacting social function, emotional well-being, physical function, and overall daily activities [2, 6, 7, 9, 10]. Bowel urgency, a symptom central to the patient experience of UC, is important to assess in UC clinical research. Bowel urgency-related accidents (i.e., accidents associated with bowel urgency) can be distinguished from other kinds of incontinence, such as passive incontinence (unawareness of the passage of stool) and fecal leakage (involuntary passing of liquid or solid stool) [11]. During UC “flares” (active or worsening symptoms), bowel urgency and bowel urgency-related accidents can become more frequent [12], which may lead to psychological distress that negatively impacts patients’ well-being [13]. Improvements in bowel urgency are associated with improvements in UC patients’ quality of life and other clinical markers of UC [14]. 

Research activities were conducted to develop and evaluate select patient-reported outcome (PRO) questionnaires for the purpose of assessing bowel urgency in different contexts of use, including a single-item Numeric Rating Scale (NRS) for urgency severity (Urgency NRS) [6], which was modified from the original 24-hour recall period item for the purposes of this work, two global assessments (Patient Global Impression of Severity [PGIS], and Patient Global Impression of Change [PGIC]), and four items of the Inflammatory Bowel Disease Questionnaire (IBDQ) related to bowel urgency and accidents (i.e., Items 11, 16, 23, 26).

The primary objectives of this paper are to present qualitative results from patient interviews to (1) document patients’ interpretation and perspective on relevance of two potential recall periods to modify the Urgency NRS for the assessment of bowel urgency associated with UC in clinical practice; (2) document the relevance of the PGIS, PGIC, and select IBDQ items to the patient experience of bowel urgency; and (3) explore the patient perspective of meaningful change in bowel urgency as assessed by the select PRO questionnaires.

## Methods

### Interview conduct

Ten 75-minute interviews were conducted via web-enabled teleconference with adults with a clinician-confirmed diagnosis of moderately to severely active UC on the Physician’s Global Assessment of UC Disease Severity between July 2022 and January 2023. Adults with UC were the target population for these interviews, and the sample size was determined based on the project’s overall goals and other pragmatic considerations.

Recruitment was conducted through an external recruitment agency that liaised with clinical sites to identify eligible patients. Patients needed to be 18 years of age or older, with a clinician confirmed diagnosis of moderate to severe UC for at least 6 months, have experienced bowel urgency within the past 6 months, fluent in English, able and willing to participate in an audio-recorded telephone interview, not have had prior surgery to treat UC, and not have a condition or situation which might put the participant at risk, confound study results, or interfere with the participant’s ability to complete the activities of the study (e.g., cognitive impairment). Patients were recruited from four clinical sites located across the United States in Chicago, Illinois; Los Angeles, California; Pittsburgh, Pennsylvania; and St. Louis, Missouri. All patients provided written informed consent to participate, as well as verbal consent at the beginning of the interview to be audio-recorded.

Trained qualitative interviewers followed a semi-structured interview guide to facilitate a discussion focused on patient interpretation of select PRO questionnaires and a hypothetical exploration of the amount of change that would be meaningful to patients on select PRO questionnaires. Descriptions of the questionnaires debriefed with patients (i.e., Urgency NRS, PGIS, PGIC, and select IBDQ items) during these interviews are presented in Table 1 (please see supplemental material Table 1 for sample questions from the qualitative interview guide).

**Table 1:** Questionnaire descriptions

Questionnaire	Item language	Description
Urgency Numeric Rating Scale (NRS) [6, 19]	Original language: • How severe was your urgency (sudden or immediate need to have a bowel movement) in the past 24 h? Modified versions used for this study: • How severe was your urgency (sudden or immediate need to have a bowel movement) in the past three days? • How severe was your urgency (sudden or immediate need to have a bowel movement) in the past seven days?	• Single-item questionnaire designed to assess bowel urgency severity over the past 24 h • Recall period: In the past 24 h – Versions with “in the past three days” and “in the past seven days” recall periods were included in this research • Response scale: 11-point NRS; 0 (No urgency)-10 (Worst possible urgency) • Scoring: Scored from 0–10; when completed as daily diary, scores averaged for 7-day periods (rounded to the nearest whole number)
Patient Global Impression of Severity (PGIS) [19]	How would you rate your overall ulcerative colitis (UC) symptoms over the past 24 h?	• Single-item questionnaire designed to assess overall disease symptom severity • Recall period: Past 24 h • Response scale: 6-point Verbal Rating Scale (VRS) (None, Very Mild, Mild, Moderate, Severe, Very Severe) • Scoring: Scored from 1–6 (1 is “None” and 6 is “Very Severe”)
Patient Global Impression of Change (PGIC) [19]	Select the response that best describes how your **UC symptoms are now**, compared to how they were before you started taking this medicine.	• Single-item questionnaire designed to assess change in global UC symptom experience over time • Recall period: Now compared to before you started taking this medicine • Response scale: 7-point VRS (Very much better, Much better, A little better, No change, A little worse, Much worse, Very much worse) • Scoring: Scored from 1–7 (1 is “Very much better” and 7 is “Very much worse”)
Inflammatory Bowel Disease Questionnaire (IBDQ) [25, 26]	Item 11: How often during the last two weeks have you been troubled because of fear of not finding a washroom? Item 16: How often during the last two weeks have you had to avoid attending events where there was no washroom close at hand? Item 23: How much of the time during the last two weeks have you felt embarrassed as a result of your bowel problem? Item 26: How much of the time during the last two weeks have you been troubled by accidental soiling of your underpants?	• 32-item disease-specific health-related quality of life questionnaire designed to assess bowel symptoms, systemic symptoms, emotional functioning, and social functioning – Items included in this research: 11, 16, 23, 26 • Recall: During the last two weeks • Response scale: Various 7-point VRSs (e.g., ranging from “All of the time” to “None of the time”) • Scoring: Response options are scored from worst “1” to best “7” • Range of possible scores from 32 to 224 (higher scores indicate better quality of life); scores between 170 and 190 may indicate patients in remission [27]

Interviews aimed to answer the following research questions: (1) Can patients interpret the updated recall periods of the Urgency NRS ([i.e., 7-day or 3-day recall period]) as intended, and provide perspective on which recall period (i.e., 7-day or 3-day) is appropriate to assess bowel urgency in a clinical practice setting?; (2) Does patient interpretation of the PGIS, PGIC, and select IBDQ items include bowel urgency and bowel urgency-related accidents?; and (3) What do patients consider to be a meaningful improvement on the Urgency NRS, PGIS, PGIC, and select IBDQ items?

During interviews, patients were asked to complete a “think-aloud” exercise (to understand patient interpretation and relevancy of PRO questionnaires of interest) and a “meaningful change” exercise (to understand what patients consider to be a meaningful improvement in their symptom experience on the PRO questionnaires of interest). During the “think-aloud” exercise [15], patients read questionnaires aloud and described what each component meant to them and the thought process they used to select responses. This exercise was utilized in order to identify words, terms, or concepts that the patient may not understand or might interpret differently than intended. During the “meaningful change” exercise, patients were asked to pick the response option for each item on the questionnaires of interest that reflected their worst experience of the item concept (i.e., the “thing” the question assesses) and then explain the smallest amount of improvement on the response scale that they would consider to be a meaningful improvement in their experience of the concept. Patients were also asked to describe how meaningful improvement on the Urgency NRS corresponded with meaningful improvement on the PGIS, PGIC, and select IBDQ items; specifically, patients were asked which PGIS, PGIC, and IBDQ response options correspond to the response they selected for their worst experience (PGIS and select IBDQ items only) and to the response they selected for the smallest meaningful improvement on the Urgency NRS.

Ethics approval was received by a centralized independent review board, Sterling Independent Review Board (IRB; IRB ID #9213), prior to interview conduct. All interviews were audio-recorded and subsequently transcribed and anonymized prior to data analysis.

### Analysis

A codebook was developed prior to beginning data analysis based on the semi-structured interview guide. Each interview transcript was “coded” via an iterative process by which researchers assign descriptive labels to interview text (single words, phrases, or longer stretches of speech) based on the study’s research questions and goals [16]. The anonymized transcripts served as the source data and were coded using qualitative coding software (ATLAS.ti Version 9) [17, 18]. 

Analysis of patient responses was conducted to assess (1) patient interpretation of the modified recall periods for the Urgency NRS and relevance of a 3-day recall period and a 7-day recall period; (2) whether patients thought of bowel urgency and bowel urgency-related accidents when interpreting the PGIS, PGIC, select IBDQ items; and (3) patient perspectives on what constitutes an important and meaningful improvement in scores on the Urgency NRS, PGIS, PGIC, and select IBDQ items.

Thematic analysis of all coded patient quotations relevant to each research question was conducted, and patients who reported similar themes were grouped together and reported by frequency. Analysis focused on patient qualitative descriptions of interpretations (i.e., what the wording of a question means to them in their own words) and of hypothetical meaningful improvements of concepts in terms of aspect (e.g., improved severity or frequency) or impact on their daily life.

Of note, patient responses were included in data analysis only if they provided sufficient information; patients who did not provide sufficient information for a given question were removed from the total denominator.

As sample size was determined based on project considerations, assessment of adequacy of the sample size via saturation analysis was deemed not critical to the analysis of this data.

## Results

### Patient characteristics

Table 2 presents the complete characteristics of the sample. A total of 10 patients completed interviews; patient ages ranged from 22.8 to 82.8 years (mean: 53.1 years [standard deviation (SD) = 16.9]). Five patients were female and five were male (*n* = 5/10, 50.0% each). Seven patients (*n* = 7/10, 70.0%) identified as White, with the remainder of patients identifying as Asian (*n* = 1/10, 10.0%), Black or African American (*n* = 1/10, 10.0%), or Hispanic (*n* = 1/10, 10.0%). Patients were recruited from one of four clinical sites: Chicago, Illinois (*n* = 5/10, 50.0%), Pittsburgh, Pennsylvania (*n* = 2/10, 20.0%), Los Angeles, California (*n* = 1/10, 10.0%), or St. Louis, Missouri (*n* = 2/10, 20.0%). Five patients (*n* = 5/10, 50.0%) were rated as having moderate disease activity by their clinicians and the remaining five patients (*n* = 5/10, 50.0%) were rated as having severe disease activity.

**Table 2:** Demographic and health characteristics of sample

Characteristic	Total sample (*N* = 10) *n* (%)
**Age**	
Range	22.8–82.8
Mean (SD)	53.1 (16.9)
**Sex** *	
Female	5 (50.0%)
Male	5 (50.0%)
**Spanish/Hispanic/Latino ethnicity** *	
Not Spanish/Hispanic/Latino	8 (80.0%)
Mexican/Mexican American, Chicano	1 (10.0%)
Puerto Rican	1 (10.0%)
**Race** *	
White	7 (70.0%)
Asian	1 (10.0%)
Black or African American	1 (10.0%)
Hispanic	1 (10.0%)
**Current living situation** *	
Living alone	6 (60.0%)
Living with family or friends (roommate, parent, child, partner/spouse)	4 (40.0%)
**Current work status** *	
Working full-time	5 (50.0%)
Retired	2 (20.0%)
Working part-time	1 (10.0%)
Student	1 (10.0%)
On disability	1 (10.0%)
**Highest level of education** *	
Some college or certificate program	5 (50.0%)
High school diploma (or GED) or less	3 (30.0%)
College or university degree (two- or four-year)	2 (20.0%)
**Severity of UC disease activity** ^**†**^	
Moderate disease	5 (50.0%)
Severe disease	5 (50.0%)
**Frequency of bowel urgency in the last week** *	
Have not experienced bowel urgency in the last week	6 (60.0%)
Several times a day	2 (20.0%)
Once or twice a day	1 (10.0%)
Once or twice a week	1 (10.0%)
**Frequency of bowel urgency-related accidents in the past four weeks** *	
Have not experienced an accident in the past four weeks	6 (60.0%)
1–4 accidents	4 (40.0%)

*Abbreviations*: *DHIF* Demographic and health information form, *IECD* Inclusion and exclusion criteria document, *SD* Standard deviation, *UC* Ulcerative colitis

*Patients reported demographic and health information via the DHIF

^†^Clinicians reported demographic and health information via the IECD

All patients (*n* = 10/10, 100.0%) met the inclusion criterion of having experienced bowel urgency within the past six months; of these, nine patients experienced bowel urgency within the past three months. Four patients (*n* = 4/10, 40.0%) reported experiencing bowel urgency within the last week at the time of screening, while six patients (*n* = 6/10, 60.0%) had last experienced bowel urgency more than a week before screening. At screening, six patients (*n* = 6/10, 60.0%) reported that they had not experienced bowel urgency-related accidents in the past four weeks, while four patients (*n* = 4/10, 40.0%) reported that they had experienced one to four accidents in the past four weeks. Patients were screened within one to 13 days prior to the day their interview was conducted.

### Evaluation of the updated recall periods for the urgency NRS

Eight patients (*n* = 8/9, 88.9%) interpreted the 7-day recall period of the Urgency NRS as intended, and one patient (*n* = 1/9, 11.1%) did not interpret the 7-day recall period as intended; instead, they interpreted it to mean the last four days. No patients suggested rewording the 7-day recall period to make it easier to understand (*n* = 9/9, 100.0%). Eight patients (*n* = 8/8, 100.0%) interpreted the 3-day recall period as intended, and no patients suggested rewording the 3-day recall period to make it easier to understand (*n* = 8/8, 100.0%).

Half of the patients (*n* = 5/10, 50.0%) reported that both the 7-day recall period and 3-day recall period were easy to understand and answer. Of the remaining patients, two patients (*n* = 2/10, 20.0%) reported that the 7-day recall period was easier to complete and three patients (*n* = 3/10, 30.0%) reported that the 3-day recall period was easier to complete. Six patients (*n* = 6/10, 60.0%) reported that the 7-day recall period was more relevant to experiences of bowel urgency because bowel urgency lasts extended periods of time or because thinking back over a week is easier cognitively; and two patients (*n* = 2/10, 20.0%) reported that the 3-day recall period was more relevant to their experience because thinking back over three days is easier cognitively. One patient (*n* = 1/10, 10.0%) reported that both the 7-day and 3-day recall periods were relevant to their experience with UC. Lastly, one patient (*n* = 1/10, 10.0%) reported that neither the 7-day nor 3-day recall period were relevant to their experience with UC; they suggested providing a range of time for the recall period instead (i.e., the past two to three days and the past four to seven days).

### Interpretation of PGIS, PGIC, and select IBDQ items relative to bowel urgency and bowel urgency-related accidents

The majority of patients reported thinking of bowel urgency (≥ 80.0%) and bowel urgency-related accidents (≥ 70.0%) when interpreting the PGIS, PGIC, and select IBDQ items. Many patients did not spontaneously describe bowel urgency or bowel urgency-related accidents in their interpretations (≥ 50.0%) of the PRO questionnaires, but rather as a result of a direct question from the interviewer (their responses were marked as “probed”). Please see Table 3 for the interpretation results and whether patients reported thinking of bowel urgency or bowel urgency-related accidents spontaneously or as a result of probing questions for all the PRO questionnaires.

**Table 3:** Patient Global Impression of Severity, Patient Global Impression of Change, and selected Inflammatory Bowel Disease Questionnaire Items interpretation summary table

Item	Interpretation concepts	n* (%)
**Patient Global Impression of Severity (PGIS)**: How would you rate your overall ulcerative colitis symptoms over the past 24 h?	**PGIS interpretation including bowel urgency**	
	Patient’s interpretation of the PGIS included bowel urgency • Spontaneous: 3/9 (33.3%) • Probed: 6/9 (66.7%)	9/9* (100.0%)
	**PGIS interpretation including urgency-related accidents**	
	Patient’s interpretation of the PGIS included urgency-related accidents • Spontaneous: 1/8 (12.5%) • Probed: 7/8 (87.5%)	8/10 (80.0%)
	Patient’s interpretation of the PGIS did not include urgency-related accidents • Probed: 2/2 (100.0%)	2/10 (20.0%)
**Patient Global Impression of Change (PGIC)**: Select the response that best describes how your ulcerative colitis symptoms are now, compared to how they were before you started taking this medicine.	**PGIC interpretation including bowel urgency**	
	Patient’s interpretation of the PGIC included bowel urgency • Probed: 10/10 (100.0%)	10/10 (100.0%)
	**PGIC interpretation including urgency-related accidents**	
	Patient’s interpretation of the PGIC included urgency-related accidents • Probed: 8/8 (100.0%)	8/10 (80.0%)
	Patient’s interpretation of the PGIC did not include urgency-related accidents • Probed: 2/2 (100.0%)	2/10 (20.0%)
**Inflammatory Bowel Disease Questionnaire (IBDQ) Item 11**: How often during the last two weeks have you been troubled because of fear of not finding a washroom?	**IBDQ Item 11 interpretation including bowel urgency**	
	Patient’s interpretation of IBDQ Item 11 included bowel urgency • Spontaneous: 7/10 (70.0%) • Probed: 3/10 (30.0%)	10/10 (100.0%)
	**IBDQ Item 11 interpretation including urgency-related accidents**	
	Patient’s interpretation of IBDQ Item 11 included urgency-related accidents • Spontaneous: 4/8 (50.0%) • Probed: 4/8 (50.0%)	8/10 (80.0%)
	Patient’s interpretation of IBDQ Item 11 did not include urgency-related accidents • Probed: 2/2 (100.0%)	2/10 (20.0%)
**IBDQ Item 16**: How often during the last two weeks have you had to avoid attending events where there was no washroom close at hand?	**IBDQ Item 16 interpretation including bowel urgency**	
	Patient’s interpretation of IBDQ Item 16 included bowel urgency • Spontaneous: 2/8 (25.0%) • Probed: 6/8 (75.0%)	8/9* (88.9%)
	Patient’s interpretation of IBDQ Item 16 did not include bowel urgency • Probed: 1/1 (100.0%)	1/9* (11.1%)
	**IBDQ Item 16 interpretation including urgency-related accidents**	
	Patient’s interpretation of IBDQ Item 16 included urgency-related accidents • Spontaneous: 1/7 (14.3%) • Probed: 6/7 (85.7%)	7/9* (77.8%)
	Patient’s interpretation of IBDQ Item 16 did not include urgency-related accidents • Probed: 2/2 (100.0%)	2/9* (22.2%)
**IBDQ Item 23**: How much of the time during the last two weeks have you felt embarrassed as a result of your bowel problem?	**IBDQ Item 23 interpretation including bowel urgency**	
	Patient’s interpretation of IBDQ Item 23 included bowel urgency • Spontaneous: 3/8 (37.5%) • Probed: 5/8 (62.5%)	8/10 (80.0%)
	Patient’s interpretation of IBDQ Item 23 did not include bowel urgency • Probed: 2/2 (100.0%)	2/10 (20.0%)
	**IBDQ Item 23 interpretation including urgency-related accidents**	
	Patient’s interpretation of IBDQ Item 23 included urgency-related accidents • Spontaneous: 2/7 (28.6%) • Probed: 5/7 (71.4%)	7/10 (70.0%)
	Patient’s interpretation of IBDQ Item 23 did not include urgency-related accidents • Probed: 3/3 (100.0%)	3/10 (30.0%)
**IBDQ Item 26**: How much of the time during the last two weeks have you been troubled by accidental soiling of your underpants?	**IBDQ Item 26 interpretation including bowel urgency**	
	Patient’s interpretation of IBDQ Item 26 included bowel urgency • Spontaneous: 5/10 (50.0%) • Probed: 5/10 (50.0%)	10/10 (100.0%)
	**IBDQ Item 26 interpretation including urgency-related accidents**	
	Patient’s interpretation of IBDQ Item 26 included urgency-related accidents • Spontaneous: 9/10 (90.0%) • Probed: 1/10 (10.0%)	10/10 (100.0%)

*Denominators are *n* < 10 due to one (*n* = 1) patient not being asked if their interpretation of the relevant item included either bowel urgency or urgency-related accidents

### Exploration of meaningful change on the urgency NRS, PGIS, PGIC, and IBDQ items

For the Urgency NRS, most patients (*n* = 8/10, 80.0%) reported a 1- to 3-point change on the 0–10 scale as the smallest level of improvement that would be meaningful; three patients (*n* = 3/10, 30.0%) each reported a 1-point and 2-point change and two patients (*n* = 2/10, 20.0%) reported a 3-point change as the smallest level of improvement that would be meaningful. One patient each (*n* = 1/10, 10.0%) reported a 7-point change and 9-point change as the smallest level of improvement that would be meaningful. Patient-reported smallest meaningful score change on the Urgency NRS, frequency of patient reporting each point change, and exemplary patient quotes, are presented in Table 4.

**Table 4:** Smallest meaningful score change on urgency numeric rating scale

Meaningful improvement	*n* (%)	Exemplary quotes describing meaningful improvement
Urgency Numeric Rating Scale (NRS): “How severe was your urgency (sudden or immediate need to have a bowel movement) in the past 24 h?” Response options: “0– No urgency,” “1,” “2,” “3,” “4,” “5,” “6,” “7,” “8,” “9,” “10– Worst possible urgency”		
Patient considers a 1-point change to be meaningful	*n* = 3/10 (30.0%)	**Patient 1 describing change from “9” to “8” on the Urgency NRS**: “Maybe less frequency, less pain, uh, less needing to find the bathroom. Not soiling as much. I mean anything is an improvement.”
Patient considers a 2-point change to be meaningful	*n* = 3/10 (30.0%)	**Patient 2 describing change from “10” to “8” on the Urgency NRS**: “Basically you’d still have to, you know, know your surroundings, but you just wouldn’t, you know, have that sudden urge of having to go 90% of the time. Maybe it’s half the time.”
Patient considers a 3-point change to be meaningful	*n* = 2/10 (20.0%)	**Patient 3 describing change from “10” to “7” on the Urgency NRS**: “That would mean a lot of not being– you know, of not having the urgency to go of not being worried about is there a bathroom close or anything of that sort.”
Patient considers a 7-point change to be meaningful	*n* = 1/10 (10.0%)	**Patient 4 describing change from “9” to “2” on the Urgency NRS**: “I feel like at a two, that would be a sufficient manageable where it’s like hey, look. I do have this but I could still lead a, a healthy, great life where I don’t really have to think about this as much as I am now.… I know it’s a big jump, but…. it just kind of messes up so many different departments of your life.”
Patient considers a 9-point change to be meaningful	*n* = 1/10 (10.0%)	**Patient 5 describing change from “10” to “1” on the Urgency NRS**: “Not having to get up in the middle of the night three, four times sometimes… not having to be worried about it and plan for it.… The medication has to feel– make the person feel comfortable that they’re in a maintain state and that’s– you know, they’re, they’re going somewhere.”

For the PGIS, eight patients (*n* = 8/10, 80.0%) reported a 1-point change as the smallest level of improvement that would be meaningful; the remaining two patients (*n* = 2/10, 20.0%) reported a 2-point change as the smallest level of improvement that would be meaningful. Patient-reported smallest meaningful score change on the PGIS, frequency of patient reporting each point change, and exemplary patient quotes, are presented in Table 5.

**Table 5:** Smallest meaningful score change on Patient Global Impression of Severity

Meaningful improvement	*n* (%)	Exemplary quotes describing meaningful improvement
Patient Global Impression of Severity (PGIS): “How would you rate your overall ulcerative colitis symptoms over the past 24 h?” Response options: “None,” “Very mild,” “Mild,” “Moderate,” “Severe,” “Very severe”		
Patient considers a 1-point change to be meaningful	*n* = 8/10 (80.0%)	**Patient 1 describing change from “Severe” to “Moderate” on the PGIS**: “The– maybe the amount of, of the pain that I experience. Uh, maybe the frequency of having to go to the bathroom. Uh, the– or maybe find a bathroom to use, uh, that certainly would be an, an improvement.” **Patient 6 describing change from “Moderate” to “Mild” on the PGIS**: “Just having like the feeling, you know, in your stomach that you have to… when you really have to go and then, you know, for whatever reason it would just happen, you know, when you have an accident sometimes or something. Just, you know, all of that and not having to, not having to avoid going somewhere because you, you, you know, can’t go to the restroom. You have this problem and they don’t even have a restroom when you really need to go.”
Patient considers a 2-point change to be meaningful	*n* = 2/10 (20.0%)	**Patient 7 describing change from “Severe” to “Mild” on the PGIS**: “Um, I would say in particular what I would think about, uh, the thing that I would want to see changed would be probably like the frequency of like stomach pains and cramps to decrease. Um, uh, a lack of like, like sudden bowel movements and urgency.… I wouldn’t have to worry too much about having to use the bathroom or experiencing like stomach pains or cramps that might like distract me from doing things that I have to get done for instance.” **Patient 8 describing change from “Very severe” to “Moderate” on the PGIS**: “Um, I think they would be somewhat less. You know, that I wouldn’t feel as– the symptoms wouldn’t be as bad. They’d be not, you know, gone but at least, you know, 25% reduction, you know.”

For the PGIC, four patients (*n* = 4/9, 44.4%) reported the response option “A little better” to be the smallest improvement that would be meaningful, and the remaining five patients (55.6%) reported the response option “Much better” as the smallest meaningful improvement. Patient-reported smallest meaningful improvement by response option on the PGIC, frequency of patient reporting each response option, and exemplary patient quotes, are presented in Table 6.

**Table 6:** Smallest meaningful improvement score on Patient Global Impression of Change

Meaningful improvement	*n* (%)	Exemplary quotes describing meaningful improvement
Patient Global Impression of Change (PGIC): “Select the response that best describes how your ulcerative colitis symptoms are now, compared to how they were before you started taking this medicine.” Response options: “Very much better,” “Much better,” “A little better,” “No change,” “A little worse,” “Much worse,” “Very much worse”		
Patient considers “A little better” to be meaningful	*n* = 4/9* (44.4%)	**Patient 1 describing the smallest meaningful improvement score on the PGIC**: “You’d see a smile on my face, as I said before. Um, just, just an overall, overall better feeling. You know, just more positive, um, wanting to get out, wanting to do things. Just, just, uh, just being happier.” **Patient 9 describing the smallest meaningful improvement score on the PGIC**: “Uh, maybe the frequency of the diarrhea. If I didn’t have that as much, that would help. And then the pain, the abdominal pain, uh, if that was lessened a little.”
Patient considers “Much better” to be meaningful	*n* = 5/9* (55.6%)	**Patient 5 describing the smallest meaningful improvement score on the PGIC**: “Um, not having to analyze a menu and decide what I can and can’t eat. Um, having to give up things you love, red meat being one of them. Um, a lot of things. And, um, having to be concerned about what social events and if you’re in a flare, you’re not going.” **Patient 7 the smallest meaningful improvement score on the PGIC**: “I would say that if my symptoms became, uh, much better I, uh, I would think about that like, um, I was experiencing like significantly less instances where, uh, I had like an urgent need to use the restroom, uh, and I was also experiencing like significantly less, uh, instances where I was experiencing like cramping or stomach pain.”

*One patient’s data were excluded because the patient did not provide sufficient data to determine which response option would represent their smallest meaningful or important improvement, resulting in a total sample of nine patients

For IBDQ Item 11, seven patients (*n* = 7/10, 70.0%) reported a 1-point change as the smallest level of improvement that would be meaningful, and the remaining three patients (30.0%) reported a 2-point change as the smallest level of meaningful improvement. For IBDQ Item 16, six patients (*n* = 6/10, 60.0%) reported a 1-point change as the smallest level of improvement that would be meaningful, three patients (30.0%) reported a 2-point change, and the remaining patient (10.0%) reported a 3-point change as the smallest level of meaningful improvement. For IBDQ Item 23, seven patients (*n* = 7/9, 77.8%) reported a 1-point change as the smallest level of improvement that would be meaningful; of the remaining two patients, one patient each (11.1%) reported a 2-point change and 3-point change as the smallest meaningful improvement. For IBDQ Item 26, six patients (*n* = 6/9, 66.7%) reported a 1-point change as the smallest level of improvement that would be meaningful, two patients (22.2%) reported a 2-point change, and the remaining patient (11.1%) reported a 3-point change as the smallest meaningful improvement. Patient-reported smallest meaningful score change on the select IBDQ items, frequency of patient reporting each point change, and exemplary patient quotes, are presented in Table 7.

**Table 7:** Smallest meaningful score change on Inflammatory Bowel Disease Questionnaire Items 11, 16, 23, and 26

Meaningful improvement	*n* (%)	Exemplary quotes^†^ describing meaningful improvement
Inflammatory Bowel Disease Questionnaire (IBDQ) Item 11: “How often during the last two weeks have you been troubled because of fear of not finding a washroom?” Response options: “All of the time,” “Most of the time,” “A good bit of the time,” “Some of the time,” “A little of the time,” “Hardly any of the time,” “None of the time”		
Patient considers a 1-point change to be meaningful	*n* = 7/10 (70.0%)	**Patient 10 describing change from “Some of the time” to “A little of the time” on the IBDQ Item 11**: “Um, going to the bathroom less frequently and having less fear of needing to find a bathroom if I’m out and about…. I mean I wouldn’t be worried about going someplace that didn’t have a restroom like, say like a– going to like a concert or something.” **Patient 3 describing change from “All of the time” to “Most of the time” on the IBDQ Item 11**: “Improvement, improvement for that would be, well like I said, most of the time would be that well it’s times where I don’t have to even think or, you know, worry about anything like that, having an accident or, you know, of that sort.” **Patient 2 change from “Some of the time” to “A little of the time” on the IBDQ Item 11**: “Um, you could be a little more flexible. You know, you could at least know that, you know, you’ve kind of got it under control. And I think in my case you would go somewhere where you can get to a toilet quickly if there was an accident.
Patient considers a 2-point change to be meaningful	*n* = 3/10 (30.0%)	**Patient 7 describing change from “Some of the time” to “Hardly any of the time” on the IBDQ Item 11**: “Uh, I would say that what would change in, in that situation would be as if, uh– I would say it would be like, I would say it would be like, um, like if I wasn’t experiencing any situations in public where, uh, I was concerned that I wouldn’t be able to find like a restroom if I was, uh, if I was experiencing any kind of like urgency that I wouldn’t be able to like, you know, control or like find a bathroom in time. Like that wouldn’t really be something that I would be worrying about in public anymore.” **Patient 8 describing change from “All of the time” to “A good bit of the time” on the IBDQ Item 11**: “Um, probably like 25% of the time it’d feel better. Like I would feel, um, not as– you know, my symptoms would be a little bit better. I’d be able to notice, you know, at least a quarter of my day would be not as completely horrible… you know, there would be less time that I would have to be focusing on that, that I could focus on other things, you know.”
**IBDQ Item 16: “How often during the last two weeks have you had to avoid attending events where there was no washroom close at hand?”**		
Patient considers a 1-point change to be meaningful	*n* = 6/10 (60.0%)	**Patient 9 describing change from “Most of the time” to “A good bit of the time” on the IBDQ Item 16**: “Uh, a good bit of the time, again it would be– these questions are kind of going to be repetitive because it would just help me get out more, kind of move a little farther from my comfort zone.” **Patient 7 describing change from “All of the time” to “Most of the time” on the IBDQ Item 16**: “Hmm, just not having to avoid going anywhere.… That would be so cool.… And now with this– with the pandemic, it’s kind of like, places don’t even let you use the restroom.”
Patient considers a 2-point change to be meaningful	*n* = 3/10 (30.0%)	**Patient 7 describing change from “Some of the time” to “Hardly any of the time” on the IBDQ Item 16**: “It would, it would indicate like a very significant improvement.… Uh, I would say that that change would indicate that I would be able to, um, I would be able to go to whatever, uh, whatever places or events that I needed to without having to really worry about or think about, uh, places where I could use the bathroom.” **Patient 4 describing change from “A good bit of the time” to “A little of the time” on the IBDQ Item 16**: “For me that would be meaningful. And it has to do a lot of what we talked about at the other questions is it’s just a significant jump where, like I said, I could live with the disease and be private about it.”
Patient considers a 3-point change to be meaningful	*n* = 1/10 (10.0%)	**Patient 5 describing change from “All of the time” to “Some of the time” on the IBDQ Item 16**: “Any degree of improvement would matter.… The goal would be to never have to avoid the event if you’re on a successful medicine. A win to me would be some of the time.… You know, if they have a medication that will, will mean that you will never have to avoid an event.… Well on a medication, I would expect– that was successful, I would expect that I would only have to avoid some of the time or a little of the time.”
**IBDQ Item 23: “How much of the time during the last two weeks have you felt embarrassed as a result of your bowel problem?”**		
Patient considers a 1-point change to be meaningful	*n* = 7/9* (77.8%)	**Patient 7 describing change from “A little of the time” to “Hardly any of the time” on IBDQ Item 23**: “Uh, I would say the feeling of embarrassment, um, probably what would change would be, uh, if I was just a little bit more confident because I didn’t have to worry about, uh, other symptoms like as much due to the treatment. I wasn’t thinking about them as much.… Uh, I would say that improvement would allow me to be, uh, more confident, uh, in public and around other people, uh, and not have to think about like any of my, uh, stomach problems or bowel problems while I’m doing things, uh, while I’m doing things that I have to do like with others.” **Patient 9 describing change from “Most of the time” to “A good bit of the time” on IBDQ Item 23**: “Again repetitively because it’s embarrassing if you soil your pants in public. So anything above that is an improvement, but I would like it to be none of the time, but I could– anything above that would be an improvement on my life.… It would help me probably expand my life, you know, how I live my life.… Uh, I would like the frequency to be down a little more.… Frequency of having to go diarrhea.”
Patient considers a 2-point change to be meaningful	*n* = 1/9* (11.1%)	**Patient 8 describing change from “All of the time” to “A good bit of the time” on IBDQ Item 23**: “Um, I would be– I would have, you know, 25% less, less time that I would have a problem with that. You know, like I would– at least a quarter of my day would not be spent, um, doing that, you know. It would– yeah.… I would, I would be able to not have to focus quite as much on that, you know.”
Patient considers a 4-point change to be meaningful	*n* = 1/9* (11.1%)	**Patient 5 describing change from “Most of the time” to “Hardly any of the time” on IBDQ Item 23**: “Um, well I’d say none of the time, but hardly any of the time, um, again because it’s a condition. And even if it’s being treated, it’s there. And I would venture to guess even on a successful medication, there will be breakthroughs or whatever.… If it’s on my mind and it very much is as, as a fear, it’s on people’s minds even more that have that experience with frequency.”
**IBDQ Item 26: “How much of the time during the last two weeks have you been troubled by accidental soiling of your underpants?”**		
Patient considers a 1-point change to be meaningful	*n* = 6/9^†^ (66.7%)	**Patient 5 describing change from “Hardly any of the time” to “None of the time” on IBDQ Item 26**: “I mean if a medication doesn’t solve that or do a very close to none of the time response, it’s, it’s not there.” **Patient 9 describing change from “Most of the time” to “A good bit of the time” on IBDQ Item 26**: “Uh, just ‘cause it’d be meaningful because I just wouldn’t– the anxiety would go down a little, which I think would affect my, uh– the frequency of it actually happening.… ’Cause I think the anxiety causes most of my– you know, it definitely is a contributing factor to what, what happens with why it happens is what– ‘cause I’m always fearful of it happening.”
Patient considers a 2-point change to be meaningful	*n* = 2/9^†^ (22.2%)	**Patient 7 describing change from “A little of the time” to “None of the time” on IBDQ Item 26**: “Uh, so for it going from a little of the time to none of the time, um, what would have changed would be if, um, would be if there were no situations in public where I was worried that, uh, I was not going to be able to like find a restroom in time and that I would have like no choice but to just like– that I wouldn’t be able to control myself. Like if there was no situations where I was like heavily concerned about that, then I would think that that would represent that change.”
Patient considers a 3-point change to be meaningful	*n* = 1/9^†^ (11.1%)	**Patient 4 describing change from “A good bit of the time” to “Hardly any of the time”**: “Um, I think it would definitely reduce stress, anxiety. It would make it easier, well in the– just the humiliation department. It would basically mean– for me, it would basically translate into yes, I have a disease. This is what I’ve got. It’s manageable. This is the– this is what I need to do to make it easier on myself and, uh, I could basically move on with my life and not have to worry about it nearly as much. So it would just basically put me at much more ease and it, and it would kind of be a domino effect in, in other departments too.”

*One patient’s data were excluded because the patient did not provide sufficient data to determine which response option would represent their worst experience, and was not asked which response option would represent the smallest meaningful or important improvement or the smallest level of meaningful change, resulting in a total sample of nine patients

^†^One patient’s data were excluded because the patient did not interpret the response scale as intended, resulting in a total sample of nine patients

Tables presenting the score changes or selected response option on the PGIS, PGIC, and IBDQ items that corresponded with the smallest level of improvement that was considered meaningful on the Urgency NRS are located in the supplemental material (supplemental material Tables 2, 3 and 4). This relationship was assessed to provide additional support for the qualitative understanding of change on the Urgency NRS by exploring corresponding meaningful changes on potential anchor items measuring related concepts.

## Discussion

Existing research has shown that bowel urgency is a prominent symptom in UC and important and bothersome to patients; [7] absence of bowel urgency is strongly associated with improvement in patient quality of life [14]. Previous work demonstrates that bowel urgency is being assessed as a key indicator of quality of life and a clinical measure of UC in the regulated clinical trial setting, and the Urgency NRS has been shown to be an appropriate bowel urgency measurement tool in adults with UC supported by documented evidence of content validity [6, 19]. In recent UC research, the patient experience of bowel urgency has been assessed using the PRO questionnaires discussed in this work in two multicenter, randomized, double-blind, placebo-controlled, Phase 3 studies to evaluate the safety and efficacy of mirikizumab for the treatment of moderately-to-severely active UC (i.e., LUCENT-1 [20] [NCT03518086] and LUCENT-2 [21] [NCT03524092] trials). This work expands on existing qualitative evidence for the Urgency NRS and also documents new qualitative patient descriptions of meaningful change in their UC and bowel urgency experience based on PRO questionnaires intended to assess those concepts.

Our findings suggested that either recall period (i.e., 3-day or 7-day) may be used with this target patient population and that patients thought both recall periods were appropriate. Evaluating patient interpretation and relevance of different recall periods indicated that the Urgency NRS may be used in different contexts; longer recall periods (i.e., 3-day or 7-days) may be more appropriate for a clinical setting than the regular 24-hour recall, as clinical practice has less frequent visits, and may be used for the purpose of informing discussions and decisions between patients and healthcare providers. Additionally, recent research was conducted to evaluate the gaps in communication between healthcare providers and patients with bowel urgency and found that bowel urgency is underappreciated by healthcare providers, despite its substantial impact on patients [22]. This further demonstrates the need for bowel urgency-specific measures that are well-suited for use in the clinical setting.

Patients reported thinking of bowel urgency when responding to the PGIS, PGIC, and select IBDQ items, therefore, these items may be appropriate as potential anchors in the estimation of meaningful change of bowel urgency in clinical trial context. Some patients spontaneously reported thinking of bowel urgency while interpreting these items; otherwise, patients were asked follow-up questions to confirm if bowel urgency was part of their interpretations. Regarding the IBDQ items, though Items 11, 16, 23 and 26 were included in this study because the concepts of measurement are closely related to bowel urgency, due to their focus on the assessment of IBD-related emotional and social functioning impacts and the two-week recall period, more patients reported thinking of bowel urgency as a result of probing rather than reported spontaneously. There were no trends in the other reported non-bowel urgency-specific symptoms or interpretations for these items, though interpretations for these items included thinking about abdominal pain, diarrhea, constipation, or thinking about whether they left the house or had social engagements at all during the last two weeks. Patients reported more frequently thinking of bowel urgency than bowel urgency-related accidents when interpreting the PGIS, PGIC, and select IBDQ items, which may reflect heterogeneity in the bowel urgency experience in the UC patient population. The lower frequency of reports of bowel urgency-related accidents among this patient population could be attributed to compensatory behaviors to avoid accidents, compared to a higher frequency of reports of bowel urgency. This is an important consideration for the development of a clinical outcome assessment measurement strategy and endpoints for clinical trials. These findings also provide insight into the patient perspective of meaningful change in the bowel urgency experience. Specifically, having patients report on the amount of change that would be meaningful to their daily lives and their UC experience across target questionnaires could be used to guide future UC research and improve healthcare providers understanding of the patient experience of UC. However, because the target questionnaires used different rating scales, meaningful change results should not be pooled or generalized across response scales. Additionally, this meaningful change exploration assumes equal distance between response options (which was not evaluated in this study); though some patient ratings of their “worst experiences” of the concept on a scale varied (e.g., the worst experience of the concept “starting” score for the meaningful change exploration for some was “Very Severe” and for others was “Very Mild”), we consider the patient’s qualitative description of their subjective experience to be trustworthy given each were able to explain why different responses were distinct and meaningful to them.

The results of this study must be interpreted in the context of certain limitations. This study’s sample size of *N* = 10 may limit the generalizability of the results of this work. However, despite the small sample size, the demographic characteristics with regard to gender, age, race and ethnicity, align with the wider UC population [23, 24]. Sample size adequacy was not assessed since sample size was determined based on pragmatic considerations; therefore, the exploratory nature of these interviews should be taken into consideration when interpreting results. Additionally, the results of the exercise to explore meaningful change to UC patients on these items was hypothetical in nature. Future work may be conducted to assess patient experience of meaningful change in a clinical trial setting to strengthen the preliminary findings of this hypothetical exercise. Although the patient perspective on meaningful change can be used to inform future research related to treatment efficacy and interpretation of scores produced by the questionnaires used in these interviews, results should be considered exploratory and may not be reflective of meaningfulness of actual changes in disease experience or questionnaires scores. Lastly, completion of each interview question with the entire sample of patients was not possible due to boundaries of the interviews, such as time constraints and the conversational nature of qualitative research (e.g., the interview guide is not a script), which, coupled with the small sample size, may also limit the generalizability of the results to the broader UC disease population.

## Conclusion

Results of these interviews serve as preliminary evidence that adults with UC interpreted the Urgency NRS with modified recall periods (i.e., 3-day or 7-day) as intended, supporting use of either modified Urgency NRS version in clinical practice settings for patients with moderate to severe UC. Additionally, these results provide evidence that most patients, when asked specifically about bowel urgency and bowel urgency-related accidents, interpreted the conceptual content of the PGIS, PGIC, and IBDQ items to be inclusive of those concepts, which signals the relevance of these PRO items to the patient experience of bowel urgency. Furthermore, these findings provide additional support to demonstrate the connection between bowel urgency, other UC symptoms, and impacts by looking at the relationship between meaningful improvements on the Urgency NRS and other PRO items. Lastly, these results demonstrated that adults with UC were able to select responses reflective of meaningful improvements in the concepts assessed by the Urgency NRS, PGIS, PGIC, and IBDQ items, which contributes additional patient-centered data to the existing UC and bowel urgency clinical research space.

### Electronic supplementary material

Below is the link to the electronic supplementary material.

Supplementary Material 1

## Data Availability

The authors confirm that relevant data supporting the findings of this study are available within the article [and/or its supplementary materials]. Additional data that support the findings of this study are available upon reasonable request from the corresponding author [THG].
