# Supplementary material for: Patient interpretations of patient-reported outcome measures to assess bowel urgency: qualitative interviews in ulcerative colitis
Source: J Patient Rep Outcomes. 2024 May 31;8:54. doi: 10.1186/s41687-024-00733-9 (PMC11143159; doi:10.1186/s41687-024-00733-9)
Supplement: Supplementary file 1 — Supplementary Material 1 [file 41687_2024_733_MOESM1_ESM.docx]

**UC Manuscript Supplemental Materials**

| Table 1. Key interview guide questions exploring meaningful change on select patient-reported outcomes |
| --- |
| **Interviewer verbal instructions to participant** |
| “Now I would like to ask you some questions about how you would know if your condition was getting better. We will be looking at the same questions we were just talking about, plus a new one that asks about severity of bowel urgency on a 0 to 10 scale. To do this, we will need to think about your worst time with UC, and then imagine you start a treatment that is working for you.” |
| **Interviewer instructions** |
| If the participant selects a response that is more than one point away from their worst experience response selected, please probe to understand whether a 1-point change would be meaningful. If the participant reports a 1-point change would not be meaningful, probe on whether a 2-point change would be meaningful, 3-point, and so on as needed. |
| **Interviewer questions to participant** |
| 1. When you are experiencing your worst symptoms, what response would you select on the [ITEM]? Could you describe why you chose that response? 2. If you were taking an effective treatment and your [ITEM CONCEPT] got better or improved, which response would represent a meaningful or important improvement to you? Why? 3. [*If the participant selects a response that is more than one point away from their worst experience response selected*]: Is that the smallest amount of change to [ITEM CONCEPT] that you would consider meaningful?    1. [*If participant selects a response that is more than one point away from their worst experience response selected*] Would an improvement of [SMALLEST IMPROVEMENT] be meaningful or important to you or not? Why?    2. [*If no*] Would an improvement of [response option that is (two points, 3 points, etc.) away from worst experience response selected] be meaningful (or important) to you or not? 4. What would have changed? (e.g., Severity? Frequency? Something else?) 5. What would this improvement mean for your daily life? |

| Table 2. Corresponding meaningful score change on the Urgency Numerical Rating Scale and Patient Global Impression of Severity (N=10) | | | | |
| --- | --- | --- | --- | --- |
| Urgency NRS smallest meaningful improvement score change^*^ | Urgency NRS smallest meaningful improvement score change  n (%) | Corresponding PGIS meaningful improvement score change^*^ | PGIS meaningful improvement score change  n (%)^‡^ | Response selected for worst experience and smallest meaningful improvement on PGIS  n (%)^§^ |
| 1 point | **Total: 3/10 (30.0%)**^†^  Response selected for worst experience on Urgency NRS “0–10”:  “9”: 1/3 (33.3%)  “7”: 2/3 (66.7%) | 0 point | 2/10 (20.0%) | “Moderate” to “Moderate”: 2/2 (100.0%) |
|  |  | 1 point | 1/10 (10.0%) | “Severe” to “Moderate”: 1/1 (100.0%) |
| 2 points | **Total: 3/10 (30.0%)**^†^  Response selected for worst experience on Urgency NRS “0–10”:  “10”: 1/3 (33.3%)  “9”: 1/3 (33.3%)  “6”: 1/3 (33.3%) | 1 point | 3/10 (30.0%) | “Moderate” to “Mild”: 1/3 (33.3%)  “Very severe” to “Severe”: 1/3 (33.3%)  “Severe” to “Moderate”: 1/3 (33.3%) |
| 3 points | **Total: 2/10 (20.0%)**^†^  Response selected for worst experience on Urgency NRS “0–10”:  “10”: 2/2 (100.0%) | 2 points | 1/10 (10.0%) | “Very severe” to “Moderate”: 1/1 (100.0%) |
|  |  | 3 points | 1/10 (10.0%) | “Very severe” to “Mild”: 1/1 (100.0%) |
| 7 points | **Total: 1/10 (10.0%)**^†^  Response selected for worst experience on Urgency NRS “0–10”:  “9”: 1/1 (100.0%) | 3 points | 1/10 (10.0%) | “Severe” to “Very mild”: 1/1 (100.0%) |
| 9 points | **Total: 1/10 (10.0%)**^†^  Response selected for worst experience on Urgency NRS “0–10”:  “10”: 1/1 (100.0%) | 4 points | 1/10 (10.0%) | “Very severe” to “Very mild”: 1/1 (100.0%) |

Abbreviations: NRS=Numerical Rating Scale; PGIS=Patient Global Impression of Severity

^*^Based on qualitative reports during the interview. Urgency NRS is scored on a 0-10 scale ranging from 0=“No urgency” to 10=“Worst possible urgency,” and PGIS is scored on a verbal response scale ranging from “None” to “Very Severe”

^†^Number of participants who reported [score change] on the Urgency NRS as reflective of the smallest meaningful improvement

^‡^Number of participants who reported [score change] on the PGIS as reflective of the smallest meaningful improvement [score change] on the Urgency NRS

^§^Number of participants who reported [response option] to [response option] on the PGIS as reflective of the worst experience to smallest meaningful improvement on the Urgency NRS

| Table 3. Corresponding meaningful score change on the Urgency Numerical Rating Scale and select Inflammatory Bowel Disease Questionnaire items | | | | | | |
| --- | --- | --- | --- | --- | --- | --- |
| Urgency NRS smallest meaningful improvement score change^*^ | Urgency NRS smallest meaningful improvement score change  n (%) | Corresponding IBDQ Item meaningful improvement score change^*^ | | IBDQ Item meaningful improvement score change  n (%)^†^ | | Response selected for worst experience and smallest meaningful improvement on IBDQ Item  n (%)^‡^ |
| **Item 11. How often during the last two weeks have you been troubled because of fear of not finding a washroom? (N=10)** | | | | | | |
| 1 point | **Total: 3/10 (30.0%)**^§^  Response selected for worst experience on Urgency NRS “0–10”:  “9”: 1/3 (33.3%)  “7”: 2/3 (66.7%) | 0 point | | 3/10 (30.0%) | | “All of the time”: 2/3 (66.7%)  “A good bit of the time”: 1/3 (33.3%) |
| 2 points | **Total: 3/10 (30.0%)**^§^  Response selected for worst experience on Urgency NRS “0–10”:  “10”: 1/3 (33.3%)  “9”: 1/3 (33.3%)  “6”: 1/3 (33.3%) | 1 point | | 2/10 (20.0%) | | “Some of the time” to “A little of the time”: 1/2 (50.0%)  “Most of the time” to “A good bit of the time”: 1/2 (50.0%) |
|  |  | 2 points | | 1/10 (10.0%) | | “All of the time” to “A good bit of the time”: 1/1 (100.0%) |
| 3 points | **Total: 2/10 (20.0%)**^§^  Response selected for worst experience on Urgency NRS “0–10”:  “10”: 2/2 (100.0%) | 2 points | | 1/10 (10.0%) | | “All of the time” to “A good bit of the time”: 1/1 (100.0%) |
|  |  | 3 points | | 1/10 (10.0%) | | “Most of the time” to “A little of the time”: 1/1 (100.0%) |
| 7 points | **Total: 1/10 (10.0%)**^§^  Response selected for worst experience on Urgency NRS “0–10”:  “9”: 1/1 (100.0%) | 4 points | | 1/10 (10.0%) | | “Most of the time” to “Hardly any of the time”: 1/1 (100.0%) |
| 9 points | **Total: 1/10 (10.0%)**^§^  Response selected for worst experience on Urgency NRS “0–10”:  “10”: 1/1 (100.0%) | 5 points | | 1/10 (10.0%) | | “All of the time” to “Hardly any of the time”: 1/1 (100.0%) |
| **Item 16. How often during the last two weeks have you had to avoid attending events where there was no washroom close at hand? (N=9)^**^** | | | | | | |
| 1 point | **Total: 2/9 (22.2%)**^§^  Response selected for worst experience on Urgency NRS “0–10”:  “9”: 1/2 (50.0%)  “7”: 1/2 (50.0%) | 0 point | 1/9 (11.1%) | | “All of the time”: 1/1 (100.0%) | |
|  |  | 3 points | 1/9 (11.1%) | | “All of the time” to “Some of the time”: 1/1 (100.0%) | |
| 2 points | **Total: 3/9 (33.3%)**^§^  Response selected for worst experience on Urgency NRS “0–10”:  “10”: 1/3 (33.3%)  “9”: 1/3 (33.3%)  “6”: 1/3 (33.3%) | 1 point | 2/9 (22.2%) | | “Some of the time” to “A little of the time”: 1/2 (50.0%)  “Most of the time” to “A good bit of the time”: 1/2 (50.0%) | |
|  |  | 3 points | 1/9 (11.1%) | | “All of the time” to “Some of the time”: 1/1 (100.0%) | |
| 3 points | **Total: 2/9 (22.2%)**^§^  Response selected for worst experience on Urgency NRS “0–10”:  “10”: 2/2 (100.0%) | 2 points | 1/9 (11.1%) | | “All of the time” to “A good bit of the time”: 1/1 (100.0%) | |
|  |  | 4 points | 1/9 (11.1%) | | “All of the time” to “A little of the time”: 1/1 (100.0%) | |
| 7 points | **Total: 1/9 (11.1%)**^§^  Response selected for worst experience on Urgency NRS “0–10”:  “9”: 1/1 (100.0%) | 4 points | 1/9 (11.1%) | | “Most of the time” to “Hardly any of the time”: 1/1 (100.0%) | |
| 9 points | **Total: 1/9 (11.1%)**^§^  Response selected for worst experience on Urgency NRS “0–10”:  “10”: 1/1 (100.0%) | 5 points | 1/9 (11.1%) | | “All of the time” to “Hardly any of the time”: 1/1 (100.0%) | |
| **Item 23. How much of the time during the last two weeks have you felt embarrassed as a result of your bowel problem? (N=10)** | | | | | | |
| 1 point | **Total: 3/10 (30.0%)**^§^  Response selected for worst experience on Urgency NRS “0–10”:  “9”: 1/3 (33.3%)  “7”: 2/3 (66.6%) | 0 point | 2/10 (20.0%) | | “Hardly any of the time”: 1/2 (50.0%) | |
|  |  |  |  |  | “A little of the time”: 1/2 (50.0%) | |
|  |  | 1 point | 1/10 (10.0%) | | “All of the time” to “Most of the time”: 1/1 (100.0%) | |
| 2 points | **Total: 3/10 (30.0%)**^§^  Response selected for worst experience on Urgency NRS “0–10”:  “10”: 1/3 (33.3%)  “9”: 1/3 (33.3%)  “6”: 1/3 (33.3%) | 1 point | 1/10 (10.0%) | | “Most of the time” to “A little of the time”: 1/1 (100.0%) | |
|  |  | 2 points | 1/10 (10.0%) | | “Some of the time” to “Hardly any of the time”: 1/1 (100.0%) | |
|  |  | 3 points | 1/10 (10.0%) | | “All of the time” to “Some of the time”: 1/1 (100.0%) | |
| 3 points | **Total: 2/10 (20.0%)**^§^  Response selected for worst experience on Urgency NRS “0–10”:  “10”: 2/2 (20.0%) | 2 points | 1/10 (10.0%) | | “All of the time” to “A good bit of the time”: 1/1 (100.0%) | |
|  |  | 4 points | 1/10 (10.0%) | | “All of the time” to “A little of the time”: 1/1 (100.0%) | |
| 7 points | **Total: 1/10 (10.0%)**^§^  Response selected for worst experience on Urgency NRS “0–10”:  “9”: 1/1 (100.0%) | 4 points | 1/10 (10.0%) | | “Most of the time” to “A little of the time”: 1/1 (100.0%) | |
| 9 points | **Total: 1/10 (10.0%)**^§^  Response selected for worst experience on Urgency NRS “0–10”:  “10”: 1/1 (100.0%) | 0 point | 1/10 (10.0%) | | “None of the time”: 1/1 (100.0%) | |
| **Item 26. How much of the time during the last two weeks have you been troubled by accidental soiling of your underpants? (N=10)** | | | | | | |
| 1 point | **Total: 3/10 (30.0%)**^§^  Response selected for worst experience on Urgency NRS “0–10”:  “9”: 1/3 (33.3%)  “7”: 2/3 (66.7%) | 0 point | 3/10 (30.0%) | | “Hardly any of the time”: 1/1 (100.0%)  “A little of the time”: 1/1 (100.0%)  “A good bit of the time”: 1/1 (100.0%) | |
| 2 points | **Total: 3/10 (30.0%)**^§^  Response selected for worst experience on Urgency NRS “0–10”:  “10”: 1/3 (33.3%)  “9”: 1/3 (33.3%)  “6”: 1/3 (33.3%) | 1 point | 2/10 (20.0%) | | “A little of the time” to “Hardly any of the time”: 1/1 (100.0%)  “Most of the time” to “A good bit of the time”: 1/1 (100.0%) | |
|  |  | 2 points | 1/10 (10.0%) | | “All of the time” to “A good bit of the time”: 1/1 (100.0%) | |
| 3 points | **Total: 2/10 (20.0%)**^§^  Response selected for worst experience on Urgency NRS “0–10”:  “10”: 2/2 (100.0%) | 2 points | 1/10 (10.0%) | | “All of the time” to “A good bit of the time”: 1/1 (100.0%) | |
|  |  | 4 points | 1/10 (10.0%) | | “All of the time” to “A little of the time”: 1/1 (100.0%) | |
| 7 points | **Total: 1/10 (10.0%)**^§^  Response selected for worst experience on Urgency NRS “0–10”:  “9”: 1/1 (100.0%) | 5 points | 1/10 (10.0%) | | “All of the time” to “Hardly any of the time”: 1/1 (100.0%) | |
| 9 points | **Total: 1/10 (10.0%)**^§^  Response selected for worst experience on Urgency NRS “0–10”:  “10”: 1/1 (100.0%) | 0 point | 1/10 (10.0%) | | “None of the time”: 1/1 (100.0%) | |

Abbreviations: IBDQ=Inflammatory Bowel Disease Questionnaire; NRS=Numerical Rating Scale

^*^Based on qualitative reports during the interview. Urgency NRS is scored on a 0-10 scale ranging from 0=“No urgency” to 10=“Worst possible urgency,” and IBDQ Items 11, 16, 23, and 23 are scored on a verbal response scale ranging from “All of the time” to “None of the time”

^†^Number of participants who reported [score change] on each IBDQ Item as reflective of the smallest meaningful improvement [score change] on the Urgency NRS

^‡^Number of participants who reported [response option] to [response option] on each IBDQ Item as reflective of the worst experience to smallest meaningful improvement on the Urgency NRS

^§^Number of participants who reported [score change] on the Urgency NRS as reflective of the smallest meaningful improvement

^**^One participant’s data were excluded as the participant did not correctly select a response option on IBDQ Item 16 to correspond to the worst experience response option, the smallest meaningful response selected, and the smallest level of meaningful change on the Urgency NRS, resulting in a total of nine participants

| Table 4. Corresponding meaningful score change on the Urgency Numerical Rating Scale and Patient Global Impression of Change table (n=8) | | |
| --- | --- | --- |
| Urgency NRS smallest meaningful improvement score change^*^ | Urgency NRS smallest meaningful improvement score change  n (%)^†^ | Corresponding PGIC meaningful improvement response selected  n (%)^§^ |
| 1 point | **Total: 2/8 (25.0%)**^‡^  Response selected for worst experience on Urgency NRS “0–10”:  “7”: 2/2 (100.0%) | “A little better”  2/8 (25.0%) |
| 2 points | **Total: 2/8 (25.0%)**^‡^  Response selected for worst experience on Urgency NRS “0–10”:  “9”: 1/2 (50.0%)  “6”: 1/2 (50.0%) | “A little better”  2/8 (25.0%) |
| 3 points | **Total: 2/8 (25.0%)**^‡^  Response selected for worst experience on Urgency NRS “0–10”:  “10”: 2/2 (100.0%) | “A little better”  2/8 (25.0%) |
| 7 points | **Total: 1/8 (12.5%)**^‡^  Response selected for worst experience on Urgency NRS “0–10”:  “9”: 1/1 (100.0%) | “Much better”  1/8 (12.5%) |
| 9 points | **Total: 1/8 (12.5%)**^‡^  Response selected for worst experience on Urgency NRS “0–10”:  “10”: 1/1 (100.0%) | “Much better”  1/8 (12.5%) |

Abbreviations: NRS=Numerical Rating Scale; PGIC=Patient Global Impression of Change

^*^Based on qualitative reports during the interview. Urgency NRS is scored on a 0-10 scale ranging from 0=“No urgency” to 10=“Worst possible urgency”

^†^Two participants’ data were excluded as the participants did not correctly select a response option on the PGIC to correspond to the smallest meaningful improvement response selected and the smallest level of meaningful change on the Urgency NRS, resulting in a total of eight participants

^‡^Number of participants who reported [response option] on the PGIC as reflective of the smallest meaningful improvement [score change] on the Urgency NRS

^§^Number of participants who reported [score change] on the Urgency NRS as reflective of the smallest meaningful improvement
